# Modifiable and non-modifiable risk factors for failure of non-operative treatment of pediatric forearm fractures: Where can we do better?

**DOI:** 10.1177/18632521231182420

**Published:** 2023-06-28

**Authors:** Nakul S Talathi, Brendan Shi, Jeremy Policht, Bailey Mooney, Kevin Y Chen, Mauricio Silva, Rachel M Thompson

**Affiliations:** 1 Department of Orthopaedic Surgery, University of California, Los Angeles, Los Angeles, CA, USA; 2 Luskin Orthopaedic Institute for Children, Los Angeles, CA, USA

**Keywords:** Closed reduction and casting, forearm fractures, pediatric distal radius fractures, pediatric trauma

## Abstract

**Introduction:**

Distal third forearm fractures are common fractures in children. While outcomes are generally excellent, some patients fail initial non-operative management and require intervention. The purpose of this study is to identify independent risk factors associated with failure of closed reduction.

**Methods:**

We conducted a retrospective review of distal third forearm fractures in children treated with closed reduction and casting. Patients were divided into two cohorts—those who were successfully closed reduced and those who failed initial non-operative management. Demographic characteristics, cast type, cast index, radiographic fracture, soft tissue characteristics, and quality of reduction were analyzed between groups.

**Results:**

A total of 207 children treated for distal third forearm fractures were included for analysis. A total of 190 (91.8%) children maintained their reduction while 17 (8.2%) failed initial non-operative management. Modifiable risk factors associated with loss of reduction on univariate analysis included the use of a long arm cast (p = 0.003), increased post-reduction displacement (p = 0.02), and increased post-reduction angular deformity (p = 0.01). Non-modifiable risk factors included increased body mass index (p = 0.02), increased presenting fracture displacement (p = 0.002), and increased width of the soft tissue envelope at the fracture site (p = 0.0001). The use of long arm casts (13% vs 2%, odds ratio = 6.44) and soft tissue width (60.6 vs 50.4 mm, odds ratio = 1.1) remained significant risk factors for loss of reduction after multivariate analysis.

**Conclusion:**

Both larger soft tissue envelope at the site of the fracture and long arm cast immobilization are independently associated with an increased risk of failing initial closed reduction in distal third forearm fractures in the pediatric population.

**Level of evidence:**

level III Case Control Study

## Introduction

The majority of pediatric forearm fractures are successfully managed with closed reduction (CR) and casting or splinting,^1,2,3^ but published success rates for CR of pediatric forearm fractures vary widely.^1–4^ Among studies that aimed to specifically investigate re-displacement following initial CR, failure rates varied between 7.3% and 21.3%.^1,4–6^ Factors that have previously been associated with fracture re-displacement after CR can be grouped into patient and injury-related characteristics (non-modifiable risk factors) such as body mass index (BMI),^7,8^ amount of initial displacement,^4,6,9^ and the presence of an ipsilateral ulnar fracture^4,5^ as well as treatment-related factors (modifiable risk factors) such as a poor cast index.^3,5^ However, these risk factors have not been re-evaluated in a large cohort in the last decade, and newer literature does not include regression analysis critical to drawing relevant conclusions from the findings. Efforts to identify contemporary, independent factors associated with an increased risk of failing initial closed treatment are important as the distal radius remains the most commonly fractured bone in children.^10^ As such, even incremental improvements in success rates can result in widespread impact. The purpose of this study was to perform a comprehensive analysis of both patient-related and treatment-related risk factors associated with re-displacement following the CR of distal third forearm fractures in a large pediatric cohort.

## Methods

An institutional review board (IRB)-approved, retrospective review of children aged 2–17 years who consecutively presented with a displaced distal third forearm fracture and who underwent CR at a dedicated pediatric orthopedic urgent care center over a 2-year period (2019–2021) was completed. Patients were included for analysis if they presented with displaced fractures of either the distal third of the radius and ulna or isolated fractures of the distal third of the radius or the ulna. Gustilo and Anderson type I open fractures and all closed fractures were included. Patients who presented with middle or proximal third forearm fractures, those who presented with multiple fractures, and those with open fractures necessitating surgical debridement were excluded from analysis. All included patients were provided either local anesthetic administered by the provider or oral pain medication at the time of attempted CR. Closed manipulation was performed by either an orthopedic advanced practice provider or a second- or fourth-year orthopedic resident with or without fluoroscopic guidance. Adequacy of reduction was determined by the treating provider (within published acceptable ranges for patient age).^11–13^ Immobilization was achieved with a plaster cast overwrapped with fiberglass. Cast length—short arm (below the elbow) or long arm (above the elbow)—was per provider’s preference, not according to fracture characteristics or patient age. Casts were applied by a licensed cast technician under the direct supervision of the primary provider with the primary provider maintaining the reduction while the cast technician applied the cast.

**Figure 1.**
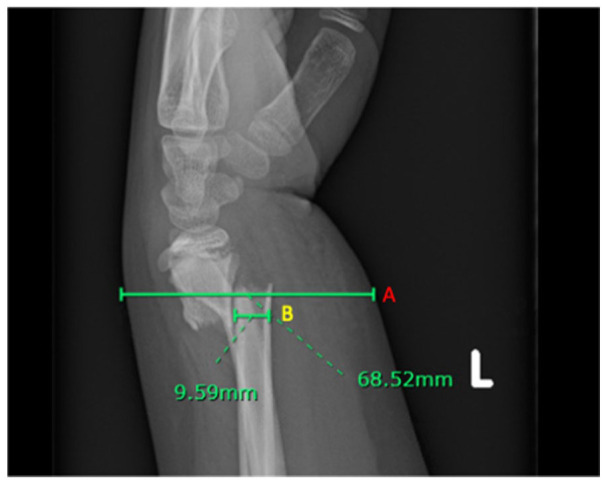
Sagittal radiograph of a displaced distal third both bone fracture in a skeletally immature patient. (A) indicates the soft tissue width at the fracture site, and (B) indicates the radius width at the fracture site.

Patients were divided into two cohorts for analysis—those whose reductions were maintained at their first follow-up visit (7–10 days post-reduction) and those who lost reduction, requiring subsequent repeat closed or open treatment. The decision to perform repeat CR or open reduction in the operating room at the time of first follow-up was at the discretion of one of five fellowship-trained pediatric orthopedic surgeons. All patients who lost reduction had a minimum of nine degrees of increase in angulation in the sagittal and/or coronal planes. Two patients were taken to the operating room (one for open reduction internal fixation and one for CR and nailing), six had casts who were wedged, and nine were re-reduced and placed into a new cast. For all patients, demographic characteristics, including age, gender, BMI, and insurance type, were abstracted from the electronic medical record. BMI was documented per the Centers for Disease Control and Prevention (CDC) guidelines for children (normal weight, overweight, and obese).^14^ Radiographic measurements included for analysis were initial angulation in the coronal and sagittal planes, initial amount of displacement measured in terms of the amount of bony overlap lost (0%–49%, 50%–99%, or 100%+), distance from the fracture to the nearest physis, and shortening at presentation. We further included a measurement of the width of the soft tissue envelope at the fracture site (Figure 1, A) and the width of the radius at the same level (Figure 1, B) both in the sagittal plane.

Post-reduction and casting measurements included whether a straight ulnar border was achieved over the length of the ulnar side of the cast, whether the appropriate mold for the pattern was applied (molded in the direction opposite of the position of injury), cast index,^15^ and quality of reduction—fair (defined as greater than 10° of residual angulation), good (defined as 5°–10° of residual angulation), or anatomic (defined as less than 5° of residual angulation). Differences in demographic and treatment variables were compared between groups.

Chi-square tests were used to compare categorical variables, Student’s T-test was used to compare parametric continuous variables, and Mann–Whitney U test was used to compare non-parametric continuous variables. Next, purposeful entry multivariate logistic regression was performed to control for confounding variables. All statistical analysis was performed on STATA software, version 12 (College Station, TX: StataCorp LLC.). P-values of 0.05 or less were deemed statistically significant.

## Results

A total of 207 children treated with CR and casting for distal third forearm fractures were included for analysis; 64 (31%) were female, and 144 (69%) were male. Seventeen (8.2%) failed to maintain initial CR and required secondary intervention (loss of reduction (LOR)); 190 (91.8%) children maintained their reduction (non-loss of reduction (non-LOR)).

Demographic characteristics are outlined in Table 1.

**Table 1.** Demographic Characteristics.

	Maintained reduction (n = 191) n (%)	Loss of reduction (n = 17) n (%)	p value
Sex			0.90
Male	132 (69%)	12 (71%)	
Female	59 (31%)	5 (29%)	
Age (mean ± SD)	9.50 ± 0.27	8.29 ± 0.68	0.19
Body mass index			0.02^a^
Normal	81 (43%)	2 (12%)	
Overweight	30 (16%)	2 (12%)	
Obese	79 (41%)	13 (76%)	
Insurance status			
Private	62 (32%)	4 (24%)	0.74
Public	117 (61%)	12 (71%)	
Uninsured	12 (6%)	1 (6%)	

SD: standard deviation.

a Indicates p < 0.05 on univariate analysis.

There was no difference in gender (p = 0.90), type of insurance (p = 0.74), or age (p = 0.19) between the LOR and non-LOR cohorts. On univariate analysis, the LOR group was found more likely to be obese (0.02), but this did not retain its significance after performing multivariate regression.

Non-modifiable, fracture-specific factors (Table 2) that were similar between the LOR and non-LOR groups included anatomic location of fracture (p = 0.36), fracture type (open vs closed) (p = 0.49), amount of shortening at the fracture site at presentation in millimeters (p = 0.26), open versus closed physes (p = 0.8), and degree of angulation at the fracture site at presentation in the coronal (p = 0.19) and sagittal (p = 0.75) planes. Those that failed to maintain initial CR (LOR) presented with significantly more displacement at the time of injury compared to the non-LOR group (p = 0.002) on univariate analysis, but this did not remain true after multivariate regression.

**Table 2.** Non-Modifiable Risk Factors.

	Maintained reduction (n = 191) n (%)	Loss of reduction (n = 17) n (%)	p value
Fracture location			0.36
Distal radius	79 (41%)	5 (29%)	
Distal radius + ulna	51 (27%)	6 (35%)	
Distal third radial shaft	41 (21%)	2 (12%)	
Distal third ulnar shaft	1 (1%)	0	
Distal third radial and ulnar shaft	19 (10%)	4 (24%)	
Fracture type			
Closed	185 (97%)	17 (100%)	0.49
Open	6 (3%)	0 (0%)	
Shortening (mm), mean ± SD	0.50 ± 1.19	1.00 ± 1.80	0.26
Physes			
Closed	5 (3%)	0 (0%)	
Open	186 (97%)	17 (100%)	0.80
Distance from fracture to physis (mm), mean ± SD	19.66 ± 15.78	24.75 ± 27.65	0.85
Displacement at injury			
0%–49%	162 (85%)	10 (59%)	0.002^a^
50%–99%	11 (6%)	5 (29%)	
100%	17 (9%)	2 (12%)	
Injury angulation (°), mean ± SD			
Coronal plane	4.64 ± 6.23	6.11 ± 7.29	0.19
Sagittal plane	16.69 ± 10.72	17.13 ± 13.54	0.75
Width of bone at fracture site (mm), mean ± SD	11.74 ± 6.69	10.39 ± 3.51	0.71
Width of soft tissue at fracture site (mm), mean ± SD	50.39 ± 9.29	60.64 ± 11.24	0.0001^a,b^

a Indicates p < 0.05 on univariate analysis.

b Indicates p < 0.05 on multivariate analysis.

Two additional non-modifiable factors that were measured at presentation were the width of the radius at the fracture site and the width of the soft tissue envelope at the fracture site, both measured in the sagittal plane (Figure 1). The bone width was consistent between the LOR and the non-LOR cohorts (p = 0.71), but the soft tissue envelope measurement was 10 mm larger, on average, in the LOR group than the non-LOR group (60.6 vs 50.4 mm, p = 0.0001). This remained significant after adjusting for confounders with multivariate regression, with an odds ratio (OR) of 1.1 (95% confidence interval (CI) = 1.01–1.2, p = 0.03).

Modifiable or treatment-specific risk factors can be found in Table 3.

**Table 3.** Modifiable Risk Factors.

	MR (n = 191) (%) n	LOR (n = 17) (%) n	p value
Cast type			
Long arm cast	97 (51%)	15 (88%)	0.003^a,b^
Short arm cast	93 (49%)	2 (12%)	
Cast index (mean ± SD)	0.71 ± 0.18	0.72 ± 0.06	0.21
Reduction quality			
Fair	62 (32%)	7 (44%)	0.04^a^
Good	16 (8%)	4 (25%)	
Anatomic	111 (60%)	5 (31%)	
Molded appropriately			
Yes	172 (90%)	16 (94%)	0.66
No	17 (10%)	1 (6%)	
Post-reduction displacement			
0%–49%	180 (95%)	14 (82%)	0.02^a^
50%–99%	6 (3%)	3 (18%)	
100%+	3 (2%)	0	
Post-reduction angulation (°) (mean ± SD)			
Coronal plane	1.25 ± 2.48	3.52 ± 6.21	0.01^a^
Sagittal plane	4.31 ± 4.59	9.7 ± 13.16	0.01^a^

MR: maintained reduction; LOR: loss of reduction.

a Indicates p < 0.05 on univariate analysis.

b Indicates p < 0.05 on multivariate analysis.

While reduction quality (p = 0.04) and post-reduction angulation in the coronal (p = 0.01) and sagittal planes (p = 0.01) were found to be significantly associated with failing CR in univariate analysis, only the use of a long arm cast (LAC) maintained significance following multivariate regression. In the LOR group, 13% of children were placed in LACs, while 2% of children in the non-LOR group were placed in LACs; as such, the use of an LAC resulted in an odds ratio of 6.4 for failing CR (95% CI = 1.3–32.7, p = 0.03). Notably, there was no difference in cast index between the LAC and the SAC cohort (0.71 vs 0.71, p = 0.99). There was no difference in cast index (p = 0.21) or application of an appropriate mold (p = 0.66) between the reduction cohorts. Table 4 provides mean angulation at reduction and at follow-up in the loss of reduction group, while Appendix Table 5 provides the specifics for each patient who lost reduction, along with their ages and treatment.

**Table 4.** Mean Changes in Angulation in the LOR Cohort.

	Mean ± SD	Range
Age (years)	8.29 ± 2.80	4–13
Reduced coronal angulation (°)	3.53 ± 6.2	0–26
Reduced sagittal angulation (°)	9.76 ± 13.16	0–58
Initial visit coronal angulation (°)	13.41 ± 10.67	0–32
Initial visit sagittal angulation (°)	18.35 ± 16.24	0–68
Coronal change (°)	–10.59 ± 8.88	–26–3
Sagittal change (°)	–7.94 ± 9.22	–23–8

LOR: loss of reduction.

## Discussion

Consistent with historic reports, our study found that CR and casting is an effective, efficient treatment for displaced distal third forearm fractures in children. Our rate of failure—8.2%—is similar to the failure rates published in other series, including reports from Hang et al. (7.3%),^4^ Voto et al. (7%),^16^ Jones et al. (7.3%),^1^ and Arora et al. (11.5%),^5^ but our study has several distinct advantages that may increase the applicability of our findings. It is a large, *consecutive cohort* of pediatric forearm fractures treated at a *single institution* with a *consistent protocol—*all included patients were treated in the urgent care setting, not the operating room, utilizing local or oral anesthetic for analgesia. Some of the landmark studies listed above and widely cited are greater than 30 years out of date, warranting this current re-investigation, which may better reflect current mechanism of injury, overall bone health, and associated outcomes in a more contemporary cohort.

Intuitively, a fracture that is more severely displaced on presentation is not reduced anatomically and demonstrates post-reduction angular deformity is more likely to fail CR. These findings have been published previously.^3–6,17,18^ And while our univariate analysis supported these results, after controlling for confounders, none of these factors were independently associated with an increased risk of loss of reduction. Rather, only factors associated directly with the ability to *maintain* a CR—use of an LAC and an increased soft tissue envelope (a measure of local adiposity and/or acute swelling)—were independently associated with an increased risk of failing initial CR. As there is a wide range of acceptable fracture parameters^11^ following CR, especially in younger children and in fractures that are in close proximity to the distal radial physis, it is unlikely that any single radiographic marker would be independently associated with failed maintenance of CR. And while previous series did find a correlation between post-reduction radiographic and casting parameters, these studies utilized general anesthesia for reduction and casting, limiting the applicability of their findings as cast placement and reduction are generally more facile under these conditions, which may result in more distinctly obvious outliers.

In contrast, in this study, after controlling for confounders, there were no significant differences in fracture displacement on presentation or after reduction between groups. There was a range of 0°–26° in post-reduction coronal angulation and 0°–25° in post-reduction sagittal angulation. The vast majority of patients had less than one half bone width of displacement. The only two factors in this series that remained significantly associated with an increased risk for loss of reduction following an initial acceptable CR after controlling for confounders were increased local soft tissue envelope and the use of an LAC, both of which contribute to the ability to *maintain* fracture position and mechanical stability at the fracture site. Perhaps, the ability to maintain fracture position is of superior importance in the successful management of these injuries rather than achieving anatomic reduction. Poor mechanical stability is more likely to lead to LOR than less-than-anatomic initial reduction.

To our knowledge, our study is the first report on the association between increased soft tissue envelope and increased risk for loss of reduction in distal third pediatric forearm fractures. The measurement we utilized was straightforward (Figure 1) and may be valuable to other providers when discussing treatment options with families. We found that patients with a 60 mm soft tissue envelope at the fracture site were significantly more likely to fail initial successful CR than those with a 50 mm soft tissue envelope. Interestingly, while this local measure of adiposity (and/or acute swelling) was highly correlated with failure, general obesity measured by BMI was *not* significantly associated with an increased risk for failing initial successful CR on multivariate analysis, which is inconsistent with findings from previous studies.^7,8^ We did find that obese patients were more likely to have larger soft tissue envelopes at the fracture site than normal weight patients (5.76 vs 4.85 mm, p = 0.003). We speculate that soft tissue envelope likely captures both local adiposity and acute swelling caused by unstable fractures, perhaps making it a reliable marker for increased risk of loss of reduction than simply BMI. Patients with more unstable fractures may present with increased initial swelling, which would contribute to a larger soft tissue envelope measurement on presentation. In this case, this swelling may decrease prior to follow-up and lead to mechanical instability and subsequent displacement. The presence of a large soft tissue envelope may have implications for timing of follow-up and discussion with families regarding likelihood of successful CR. Patients with greater soft tissue envelopes should be counseled on a possible higher risk of losing initial reduction. Future research is warranted to determine the effect of dynamic swelling as opposed to stable local adiposity on fracture stability. We additionally found that the only *modifiable* risk factor associated with failing CR in this cohort was the use of an LAC. Short arm casts have long been found to be as effective in treating distal third forearm fractures, with ample evidence to suggest there is no difference in outcomes between long and short arm casts.^3,18^ Our results are consistent with those of Webb’s randomized control trial,^17^ who also found that patients treated with an LAC were more likely to fail CR. However, all the failed CRs in that series had poor cast indices, regardless of cast length/design. At our institution, our cast technicians apply long arms in a single application as opposed to converting an initial SAC to an LAC. We agree with their hypothesis that LACs may be more technically challenging to apply, especially with a good cast index and appropriate mold. That said, in our series, both the non-LOR and LOR cohorts had similar cast indices, well within acceptable parameters (0.71 vs 0.72, p = 0.21) and there was no difference in CI between the LAC and SAC cohort (0.71 vs 0.71, p = 0.99), perhaps allowing us to better isolate the effects of cast length on failure rates, although the decision to use an LAC versus SAC was not controlled for and remains a potential confounder. Further investigation is necessary, but our findings, along with the current body of evidence, support the use of short-arm immobilization for this fracture location, especially when taking into consideration the conveniences inherent with an SAC.

Our study is not without limitations. All fractures were treated in an urgent care rather than an emergency room setting, perhaps introducing some bias in patient selection. Our cohort was somewhat heterogeneous in chronologic age, physeal status, and mechanism of injury, although none of these factors were associated with failure of maintenance of CR. The use of LAC was at the discretion of the provider, and several factors may have played a role in selection of cast type, including fracture stability and/or provider preference. Our retrospective analysis is limited in identifying these factors as they were not documented, and a well-designed randomized controlled trial (RCT) may better elucidate the role of an LAC. Institutionally, we do not typically place more displaced fractures in LAC by rule. In addition, all of the casts were plaster casts overwrapped with fiberglass applied by experienced cast technicians, who may not be available at all centers. There were a number of cast technicians and providers involved in the reduction and casting of these patients as well, adding variability in technical skill. Long-term follow-up and the subsequent clinical impact of initial loss of reduction were not available for this study as our primary endpoint was loss of reduction at the initial follow-up appointment. Therefore, the long-term clinical impact of a failed initial CR is unknown. Generally, in this patient population, follow-up tends to be limited as patients typically are graduated from scheduled appointments after their cast is removed and there is evidence of radiographic healing, typically at 6–8 weeks post-injury. Future studies including long-term follow-up are necessary to determine the clinical implications of early loss of initial reduction. Finally, all the limitations inherent to retrospective reviews, including incomplete data abstraction, lack of blinding, and coding inconsistencies, are applicable to this study.

Despite these limitations, local soft tissue envelope—a measure of local adiposity and/or local transient swelling—and the use of an LAC are independent risk factors for failing initial CR in distal third forearm fractures in children. Given these associations, we recommend consideration of use of short arm immobilization following CR for these fractures. Furthermore, clinicians should be cognizant of larger soft tissue envelopes at the fracture site, counsel patients appropriately, and consider earlier post-reduction follow-up for those with this finding.

## Supplemental Material

sj-pdf-1-cho-10.1177_18632521231182420 – Supplemental material for Modifiable and non-modifiable risk factors for failure of non-operative treatment of pediatric forearm fractures: Where can we do better? Click here for additional data file. Supplemental material, sj-pdf-1-cho-10.1177_18632521231182420 for Modifiable and non-modifiable risk factors for failure of non-operative treatment of pediatric forearm fractures: Where can we do better? by Nakul S Talathi, Brendan Shi, Jeremy Policht, Bailey Mooney, Kevin Y Chen, Mauricio Silva and Rachel M Thompson in Journal of Children’s Orthopaedics

## Appendix

**Table 5.** Patient-Specific Fracture Angulation Changes in the LOR Cohort.

	Age (years)	Reduction coronal angulation (°)	Reduction sagittal angulation (°)	Initial visit coronal angulation (°)	Initial visit sagittal angulation (°)	Coronal change (°)	Sagittal change (°)	Treatment received
Patient 1	10	3	14	0	24	3	–10	Re-reduction
Patient 2	12	0	0	1	18	–1	–18	Open reduction, plate fixation
Patient 3	6	0	13	10	28	–10	–15	Re-reduction
Patient 4	7	1	2	7	12	–6	–10	Wedging
Patient 5	9	5	6	30	0	–25	6	Re-reduction
Patient 6	9	2	0	11	9	–9	–9	Wedging
Patient 7	10	0	10	0	21	–11	0	Re-reduction
Patient 8	4	5	5	15	28	–10	–23	Re-reduction
Patient 9	8	6	9	26	1	–20	8	Wedging
Patient 10	7	1	6	12	9	–12	–3	Wedging
Patient 11	4	26	58	26	68	0	–10	Closed reduction, intermedullary nails
Patient 12	13	1	6	20	7	–19	–1	Re-reduction
Patient 13	7	6	11	32	32	–26	–21	Re-reduction
Patient 14	6	0	5	20	0	–20	5	Re-reduction
Patient 15	10	0	13	0	22	0	–9	Wedging
Patient 16	6	0	4	10	18	–10	–14	Wedging
Patient 17	13	4	4	8	15	–4	–11	Re-reduction

LOR: loss of reduction; CR: closed reduction.
